# Assessing the relationship between pregravid body mass index and risk of adverse maternal pregnancy and neonatal outcomes: prospective data in Southwest China

**DOI:** 10.1038/s41598-021-87135-9

**Published:** 2021-04-07

**Authors:** Yue Chen, Ke Wan, Yunhui Gong, Xiao Zhang, Yi Liang, Xiaoyu Wang, Ping Feng, Fang He, Rong Zhou, Dagang Yang, Hong Jia, Guo Cheng, Toshio Shimokawa

**Affiliations:** 1 West China School of Public Health and West China Fourth Hospital, Sichuan University, Chengdu, People’s Republic of China; 2 Graduate School of Information Science and Technology, Hokkaido University, Hokkaido, Japan; 3 West China Women’s and Children’s Hospital, Sichuan University, Chengdu, People’s Republic of China; 4 Department of Clinical Nutrition, Affiliated Hospital of Guizhou Medical University, Guiyang, People’s Republic of China; 5 Department of Epidemiology and Biostatistics, School of Public Health, Southwest Medical University, Luzhou, People’s Republic of China; 6 Laboratory of Molecular Translational Medicine, Center for Translational Medicine, Key Laboratory of Birth Defects and Related Diseases of Women and Children (Sichuan University), Ministry of Education, Department of Pediatrics, West China Second University Hospital, Sichuan University, Chengdu, No.16, Section 3, Renmin Nan Road, Chengdu, 610041 Sichuan People’s Republic of China; 7 Department of Medical Data-Science, Graduate School of Medicine, Wakayama Medical University, Wakayama, Japan

**Keywords:** Epidemiology, Paediatric research, Obesity, Medical research

## Abstract

The relevance of pregestational body mass index (BMI) on adverse pregnancy outcomes remained unclear in Southwest China. This study aimed to investigate the overall and age-category specific association between pre-gestational BMI and gestational diabetes mellitus (GDM), preeclampsia, cesarean delivery, preterm delivery, stillbirth, macrosomia, and small-for-gestational age (SGA) or large-for-gestational age (LGA) neonates in Southwest China. Furthermore, it explores the relative importance of influence of pregravid BMI and maternal age on pregnancy outcomes. 51,125 Chinese singleton pregnant women were recruited as study subjects. Multiple logistic regression models were used to examine the influence of pre-pregnancy BMI on adverse pregnancy outcomes. Gradient boosting machine was used to evaluate the relative importance of influence of pregravid BMI and maternal age on pregnancy outcomes. It is found that women who were overweight or obese before pregnancy are at higher risk of adverse pregnancy outcomes except for SGA neonates, while pre-pregnancy underweight is a protective factor for GDM, preeclampsia, cesarean delivery, macrosomia and LGA, but not SGA. Younger mothers are more susceptible to GDM and macrosomia neonates, while older mothers are more prone to preeclampsia. Pre-pregnancy BMI has more influence on various pregnancy outcomes than maternal age. To improve pregnancy outcomes, normal BMI weight as well as relatively young maternal ages are recommended for women in child-bearing age.

## Introduction

For decades, the prevalence of overweight or obesity in the Chinese adult population has been increasing^1,2^. Meanwhile, the bodyweight of women in childbearing age has also increased^3^. Accumulated evidence shows that pregestational overweight or obesity may increase the risk of adverse pregnancy outcomes^4–7^. Several large prospective studies have been performed in China^6,7^. One considered 536,098 pregnant women in rural China, and found significant relationships between pre-pregnancy body mass index (BMI) and five adverse outcomes in their children^6^. In another analysis of 14,451 women in Beijing city (0.02 million km^2^, 13.5 million residents^8^), pre-pregnancy BMI was shown to be related to seven adverse maternal outcomes^7^. However, few studies have focused on Southwest China. Furthermore, previous studies have explored the effect of elevated pregravid BMI on single adverse pregnancy outcome^9^ or particular outcomes, such as maternal complications^10^ and neonatal outcomes^4^, but only few have examined pregnancy outcomes on both maternal and neonatal sides with the risk of increased BMI^11^, especially in Southwest China.

Recently, there has been a noticeable rise in the maternal age of Chinese women^12^. A nationwide descriptive comparative study in China suggested that since the announcement of the universal two child policy, women giving birth in China have been more likely to be aged 35 and over^13^. Meanwhile, adverse pregnancy outcomes are reportedly related to increasing maternal age^14^. Elevated pre-pregnancy BMI was found to increase the risk of gestational diabetes mellitus (GDM), particularly in advanced maternal age^15^. Whether maternal age have any effects on the relationships between pre-gestational maternal BMI and various adverse pregnancy outcomes, however, remained unclear.

Thus, the aim of this study is to investigate whether pre-gestational BMI is associated with GDM, preeclampsia, cesarean delivery in mothers, and preterm delivery, stillbirth, macrosomia, small-for-gestational age (SGA) and large-for-gestational age (LGA) neonates. The study also examines the potential impact of maternal age as well as the relative influence of pregravid BMI and maternal age on selective pregnancy outcomes.

## Results

General characteristics of the participants are shown in Table 1. The mean maternal age of the 51,125 women is 30.7. 22.6% of the women are categorized as overweight or obese based on pre-pregnancy BMI and 15.3% being underweight before pregnancy. Around one fifth of the participants developed GDM, and 1.6% had preeclampsia during pregnancy. Notably, up to 58.1% of the participants gave birth by cesarean section. After exclusion of 588 (1.2%) stillborn births, of the rest 51,044 live births, 51.9% were girls. 5.2% neonates were delivered preterm. Around one fifth of the children developed intrauterine overgrowth such as macrosomia (4.1%) and LGA (14.5%). Moreover, 3.3% of the children were SGA infants.

**Table 1:** General characteristics of the study participants

Characteristics	Values
**Mothers (n)**	51125
Maternal age (years)	30.7 (4.2)
Primiparous (n (%))	34876 (68.2)
Single mother (n (%))	1380 (2.7)
**Pregravid BMI**^**a**^** (kg/m**^**2**^**)**	21.1 (2.7)
Normal weight	31787 (62.2)
Under weight	7805 (15.3)
Overweight or obese	11533 (22.6)
Gestational weight gain (kg)	13.1 (4.2)
Gestational diabetes mellitus^b^ (n (%))	10361 (20.3)
Preeclampsia (n (%))	804 (1.6)
Cesarean delivery (n (%))	29720 (58.1)
**Offspring (n)**	51044
Sex, female (n (%))	26506 (51.9)
Preterm delivery (n (%))	2645 (5.2)
Stillbirth (n (%))	588 (1.2)
Gestational age at delivery (weeks)	39.0 (1.9)
Birth weight (kg)	3.3 (0.5)
Birth length (cm)	49.4 (2.6)
Macrosomia^c^ (n (%))	2104 (4.1)
Small for gestational age (n (%))	1691 (3.3)
Large for gestational age (n (%))	7423 (14.5)

Values are means (SD) or frequencies.

^a^Pregravid body mass index, categorized by WHO Asian^29^.

^b^Diagnosed by IADPSG criteria^37^.

Table 2 presents pregravid BMI characteristics of the participants. The majority of mothers gave birth between the ages of 25 and 35 (76.5%); only 4.6% born children when they were younger than 25. Compared with mothers younger than 30 when they delivered, those over 30 show an opposite pregravid BMI prevalence trend: the prevalence of being underweight before pregnancy decreased with increasing maternal age.

**Table 2:** Pregravid BMI characteristics of the study participants.

Maternal age category	Pregravid BMI category according to WHO Asian				
	Overall (n = 51,125)	Normal weight (n = 31,787)	Underweight (n = 7805)	Overweight (n = 10,334)	Obese (n = 1199)
< 25 years	2336 (4.6)	1324 (4.2)	698 (8.9)	280 (2.7)	34 (2.8)
25–29 years	19,601 (38.3)	12,409 (39.0)	3943 (50.5)	2933 (28.4)	316 (26.4)
30–35 years	19,513 (38.2)	12,304 (38.7)	2460 (31.5)	4253 (41.2)	496 (41.4)
> 35 years	9675 (18.9)	5750 (18.1)	704 (9.0)	2868 (27.8)	353 (29.4)

Odds ratio (OR) for maternal and neonatal outcomes by pre-gestational BMI categories are shown in Table 3. Adjusted for potential confounders, there were significant relationships between pre-pregnancy BMI and adverse maternal outcomes such as GDM, preeclampsia and cesarean delivery. Compared with those in normal weight category, women with pre-pregnancy overweight or obesity are at higher risk of GDM (OR 2.16), preeclampsia (OR 2.89) and cesarean delivery (OR 1.58). Meanwhile, women with pre-gravid underweight are at decreased risk of GDM (OR 0.62), preeclampsia (OR 0.64) and cesarean delivery (OR 0.66). Infants with mothers categorized as overweight or obese based on pregravid BMI are at higher risk of preterm delivery (OR 1.38), stillbirth (OR 1.69), macrosomia (OR 1.92) and LGA (OR 1.80), but at lower risk of SGA (OR 0.65). Likewise, children with underweight mothers have a lower risk of neonatal macrosomia (OR 0.40), LGA (OR 0.60), but increased risk for SGA (OR 1.74).

**Table 3:** Odds ratio for maternal and neonatal outcomes by pre-gestational BMI categories.

Adverse pregnancy outcomes	Categories of pre-gestational BMI			P^a^
	Normal weight (n = 31,787)	Under weight (n = 7805)	Overweight or obese (n = 11,533)	
**Maternal outcomes**				
Gestational diabetes mellitus^b^	1	0.62 (0.57, 0.66)	2.16 (2.05, 2.26)	< 0.001
Preeclampsia^b^	1	0.64 (0.48, 0.84)	2.89 (2.50, 3.35)	< 0.001
Caesarean delivery^c^	1	0.66 (0.62, 0.69)	1.58 (1.51, 1.65)	< 0.001
**Neonatal outcomes**				
Preterm delivery^c^	1	1.04 (0.93, 1.17)	1.38 (1.26, 1.51)	< 0.001
Stillbirth^c^	1	1.00 (0.78, 1.28)	1.69 (1.41, 2.02)	< 0.001
Macrosomia^d^	1	0.40 (0.33, 0.48)	1.92 (1.75, 2.11)	< 0.001
Small for gestational age^d^	1	1.74 (1.54, 1.95)	0.65 (0.56, 0.75)	< 0.001
Large for gestational age^d^	1	0.60 (0.55, 0.65)	1.80 (1.70, 1.90)	< 0.001

^a^Data are OR with 95% confidence intervals. Normal weight group is the reference group. Logistic regression analysis was applied to calculate adjusted ORs, and Wald’s test was used.

^b^Model adjusted for maternal age, gestational weight gain, parity, gestational age, infant’s sex, marital status.

^c^Model adjusted for maternal age, gestational weight gain, parity, gestational age, infant’s sex, marital status, gestational diabetes mellitus, preeclampsia.

^d^Model adjusted for maternal age, gestational weight gain, parity, gestational age, infant’s sex, marital status, gestational diabetes mellitus.

**Figure 1:**
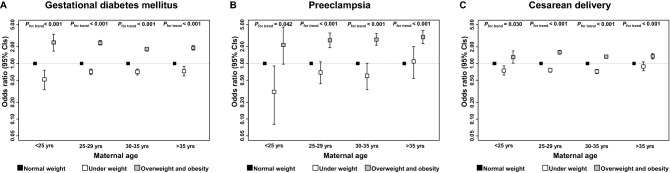
Pre-gestational BMI and risks of maternal pregnancy outcomes stratified by maternal age. Figures (**A**–**C**) show the age specific odds ratio and 95% CI for pre-pregnancy BMI on maternal outcomes. Models were adjusted for maternal age, gestational weight gain, parity, gestational age, infant’s sex and marital status, and models in (**C**) were additional adjusted for GDM and preeclampsia.

The risks of underweight, overweight, or obese pre-pregnancy on maternal pregnancy outcomes are exhibited in Fig. 1, stratified by maternal age. The significant association between elevated pre-gestational BMI and higher risk of GDM exist across all age ranges, but was much more evident in mothers younger than 30 years. Within the higher age categories, there is an increasing trend in the risk of pre-pregnancy overweight or obesity on preeclampsia. The protective effect of underweight, however, was merely significant in mothers of the youngest age category. Despite the age categories, pregravid overweight or obesity are risk factors for conducting a cesarean delivery, but the odds ratio peaked in mothers between 25 and 29. Apart from women of advanced maternal age (> 35), women who was underweight before bearing children were least likely to undergo cesarean delivery.

**Figure 2:**
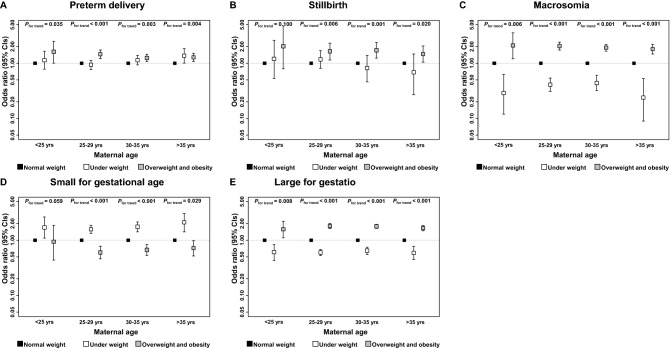
Pre-gestational BMI and risks of infant outcomes stratified by maternal age. Figures (**A**–**E**) show the age specific odds ratio and 95% confidence intervals for pre-pregnancy BMI on infant outcomes. Models were adjusted for maternal age, gestational weight gain, parity, gestational age, infant’s sex, marital status, GDM, and models in (**A**,**B**) were additionally adjusted for preeclampsia.

Figure 2 displays risks of pre-pregnancy underweight, overweight, or obesity on infant outcomes, stratified by maternal age. With higher maternal age, there is a slightly decreased trend in the risk of higher pre-pregnancy BMI on preterm delivery, and its odds ratio reached a low point at the maternal age of 30–35. Meanwhile, categorized as underweight based on pre-pregnancy BMI is also a risk factor for preterm delivery in the highest maternal age category. Across all maternal age categories, there are positive associations between increasing pre-gestational BMI and macrosomia and LGA. Negative associations were observed between pre-gestational BMI and SGA in infants whose mother’s age was over 25. Children with pre-pregnancy overweight or obese mothers were at higher risk of stillbirth in the same maternal age categories.

As shown in Table 4, pregravid BMI indicates more influence on GDM, preeclampsia, stillbirth, macrosomia, SGA and LGA, while maternal age is more relevant on cesarean delivery. After further examination as explanatory variable, preeclampsia has the most significant influence on preterm delivery compared to pre-pregnancy BMI, maternal age and GDM.

**Table 4:** Variable importance using Gradient Boosting Machine.

Adverse pregnancy outcomes	Variable importance			
	Pregravid BMI	Age	GDM^a^	Preeclampsia
**Maternal outcomes**				
GDM^a^	100.0	60.3	–	–
Preeclampsia	100.0	12.2	–	–
Cesarean delivery	32.2	100.0	0.5	3.8
**Neonatal outcomes**				
Preterm delivery	17.2	29.9	11.3	100.0
Stillbirth	100.0	45.7	20.2	46.7
Macrosomia	100.0	2.3	0.6	0.2
Small for gestational age	100.0	8.6	0.3	52.3
Large for gestational age	100.0	8.9	2.0	0.4

Gradient Boosting Machine (GBM) is one of the statistical machine learning methods with the best prediction accuracy proposed by Friedman (2001)^36^. The variable importance is a measure expressed in a relative scale, when most influence variable for adverse pregnancy outcome is set to 10.

^a^Gestational diabetes mellitus.

## Discussion

The results show that women with pre-pregnancy overweight or obesity are at higher risk of GDM, cesarean delivery, preeclampsia, preterm delivery, stillbirth, and delivering macrosomia or LGA neonates. The protective effect of underweight, however, are only clear in certain maternal age categories. Furthermore, pre-pregnancy BMI has more influence on pregnancy outcomes than maternal age. Several trends that were observed in this study and their respective hypotheses are discussed below.

GDM is related to higher pre-pregnancy BMI, which was in line with the conclusion of a meta-analysis that used data from low- and middle- income countries^16^. The risk effects of elevated pre-pregnancy BMI on GDM seems to be weaker in Chinese populations than in Western populations. In an Australian cohort^17^, the odds for overweight or obesity were 2.7 and 6.5 respectively, which was 4.25 and 6.28 in a Dutch cohort^11^, whereas two Chinese cohorts displayed 1.91^7^ and 2.19^18^ odds ratios for pre-gravid overweight or obesity on GDM, corresponding with 2.16 in the current study. Furthermore, the risk effect of pre-gestational overweight or obesity on GDM was almost 1.5-fold in mothers under 30 compared to older ones. Normally, increase in both age and pre-pregnancy BMI result in higher risks of developing GDM^19^. In this study’s population, however, the risk effect of pregravid overweight or obesity on GDM decreased with increasing maternal age. A possible explanation might be that there was a contrary distribution of pre-gravid BMI categories on mothers with maternal age < 30 or > 30 in the population.

Regarding preeclampsia, there is an increased risk of elevated maternal BMI in mothers with maternal age > 25. This is probably because of the relatively small proportion of participants under 25 (4.6%).

Over half of the women in this study gave birth by cesarean deliveries, which is dramatically higher than that in Western countries (12.3% in Netherlands^11^, 19% in an Australian cohort^17^ and 22% in an American birth cohort^4^). Since information of pregnancy outcomes were derived from the Medical Birth Registry, it was unclear under which circumstances participants underwent cesarean deliveries. Besides, the protective impact of pregravid underweight was only pronounced in women with maternal age under 35, indicating a better preventive effect for maternal complications of weight management in younger mothers.

Pre-pregnancy BMI is strongly associated with neonatal outcomes^20^. Some^10,21^ but not all^22^ cohort studies found that pre-gestational BMI is positively correlated to higher risk of preterm delivery. Both pre-pregnancy overweight or obesity as well as underweight could result in higher risk of preterm delivery in advanced maternal age in this study’s population. Pre-pregnancy overweight or obesity is a risk factor for stillbirth as well. This might be due to the altered lipid metabolism in women with obesity that may cause a reduction in prostacyclin secretion and increase thromboxane production^23^. This increases the risk of placental thrombosis^24^, a common cause of stillbirth^25^.

A slightly decreased trend of risk for pre-pregnancy BMI on macrosomia was found in older mothers. In consideration of higher maternal age and its likelihood of adverse pregnancy outcomes^26^, both mothers and gynecologists pay increased attention to maintaining healthy pregnancies compared to younger mothers who are less likely to develop such outcomes. The risk of developing macrosomia may thus decrease with the increasing maternal age.

Furthermore, this study explored and quantified the relative importance of the influence of pregnancy BMI and maternal age on each pregnancy outcomes. It is found that maternal age, instead of pre-gestational BMI, has the most significance on cesarean delivery, while the latter has more influence on the other seven pregnancy outcomes. Moreover, since gestational glucose status and blood pressure play important roles in pregnancy-related endocrine homeostasis, the importance of GDM and preeclampsia were further checked with respect to cesarean delivery and neonatal birth outcomes. Previous evidence has suggested relevance of gestational diabetes on various pregnancy outcomes^27^. However, this study found the influence of GDM on birth outcomes is far less obvious than maternal age, preeclampsia, and especially pregravid BMI. Interestingly, though the direct influence of pregravid BMI on preterm delivery is not high, preeclampsia, one of the pregnancy outcomes influenced mostly by pre-pregnancy BMI, has the most influence for preterm delivery, which indicates that pregravid BMI has correlational influence on preterm delivery.

For stillbirth, though maternal age has a relatively important impact, the influence of pregravid BMI, which combined the impact from pre-gestational BMI itself and preeclampsia together, is almost threefold to the influence from maternal age. Pregestational BMI, on one hand, has an original prominent influence on GDM, preeclampsia, stillbirth, macrosomia, SGA and LGA, and on the other, has an indirect influence on preterm delivery.

To facilitate their career and family life, despite gynecologist’s advice of initiating reproduction at a younger age, modern women tend to have children at an older age^26^. Therefore, one limitation of this analysis is the relatively small proportion of mothers with maternal age < 25, and thus the lower statistical power to find accurate results. Another important limitation lays on the pre-gestational BMI calculated by self-reported pregravid weight. As women participants weren’t recruited until they are found pregnant, pre-pregnant weight was hard to be accurately measured, which might cause the recall bias to this analysis. Besides, cesarean deliveries performed under any condition was taken into account in this study. The potential confounding of different modes of cesarean delivery might interfere its association with pregravid BMI. Furthermore, the information of level of glucose control after GDM diagnoses was also unavailable, which might have caused some confounding effect to this analysis. As an advantage, both overall and age category-specific relevance between maternal BMI and pregnancy outcomes were looked at as one previous meta-analysis suggested^28^, as well as originally explored the relative influence of pregravid BMI and maternal age on selective outcomes. Some new ideas have been suggested for modern women to consider regarding appropriate timing for both positive pregnancy outcomes and better careerdevelopment.

In summary, a mother who is underweight or overweight/obese before a pregnancy could result in higher risk of adverse maternal and neonatal outcomes. To improve pregnancy outcomes, normal BMI weight as well as relatively young maternal ages are recommended before women plan to have children.

## Methods

### Study design and population

A prospective cohort study initiated in 2013 was conducted in Southwest China (Sichuan province, Yunnan province and Guizhou province, 0.49 million km^2^, 144.1 million residents^8^) to investigate the relevance of maternal nutrition status before and during pregnancy, such as pre-pregnancy BMI and gestational weight gain, for maternal and neonatal health outcomes. A representative sample of pregnant women and their children were recruited from community health care centers and public hospitals using a sampling design stratified by urban and rural locations in Southwest China. Two or three community health care centers or public hospitals were randomly selected within each location. The study was approved by the Ethics Committee of the Sichuan University, and all methods used in this study were performed in accordance with the relevant guidelines and regulations. All participants provided written informed consent for all examinations as well as for linkage of their data from the Medical Birth Registry.

Pregnant women who had lived in their current residence for at least 2 years were invited to participate in the study at their first routine ultrasound examination in gestational week 9–11. Overall, data collection was conducted three times [the first routine ultrasound examination (phase 1 (P1), gestational weeks 20–22 (P2) and gestational weeks 33–35 (P3)]. At P1, each woman was asked to complete a self-administered questionnaire on birth history and demographic characteristics. Details on anthropometric measures, clinical measures, as well as information on current and past pregnancy outcomes recorded in the Medical Birth Registry were linked to the study database.

For the current analysis, maternal pre-pregnancy anthropometry and pregnancy outcomes derived from the Medical Birth Registry were used to investigate the relationship between maternal pregravid BMI and adverse pregnancy outcomes. Only singleton pregnant mothers and their children were included in this analysis since mothers who experienced a twin pregnancy have different pregnancy metabolism. 52,221 pregnant women without diabetes mellitus or hypertension before pregnancy were recruited between 2013 and 2018. After the exclusion of 1096 participants with missing value of GDM, preeclampsia, stillbirth, infant's sex and pre-gestational BMI, 51,125 women and their children formed this study’s final sample.

### Maternal demographic characteristics and other information

A self-administered questionnaire was used to collect information on age, residence, occupation, education level and personal income of the participants at P1. In addition, reproductive history, medical history, and family history of chronic diseases were assessed by this questionnaire. Gestational age (GA) was assessed during the first ultrasound scan (Eub 5500, Hitachi; Eub 7500, Hitachi; Logiq E9, GE) on the day of registration. GA was estimated by combining ultrasonography data with self-reports on the last menstrual period. If both measures were available and they were in agreement (± 14 days) then self-reported data were used, otherwise ultrasound data were used.

### Measurement of pre-pregnancy BMI

Weight before pregnancy were self-reported by mothers, while weight during pregnancy was measured with an ultrasonic meter (Dingheng, Zhengzhou, China) to the nearest 100 g at enrollment and at regular intervals (every 4 weeks from enrollment to week 25, every 2 weeks until week 33, and weekly thereafter until birth). Pregravid height was measured with a studio meter to the nearest 0.1 cm. Pre-pregnancy BMI was calculated as weight/height^2^ (kg/m^2^). It was categorized according to WHO BMI categorization criteria recommended for Asian population^29^, in which BMI of < 18.5 kg/m^2^ BMI is recommended as underweight, 18.5–22.9 kg/m^2^ is normal weight, 23–24.9 kg/m^2^ is overweight and ≥ 25 kg/m^2^ is obese. Since only 2.3% pregnant women in the study were obese before pregnancy, overweight or obese categories were merged in the analysis.

### Measurement of maternal outcomes

Information on maternal adverse pregnancy outcomes, such as GDM, preeclampsia and cesarean delivery, were derived from the medical birth registries. Examinations and diagnoses of pregnancy outcomes were conducted by trained doctors from each study center according to acknowledged criteria. GDM was diagnosed from 2-h 75-g oral-glucose-tolerance tests at 24–28 weeks of gestation. Patients of GDM was identified if at least one of the following plasma glucose levels was met: 0 h (fasting), ≥ 5.1 mmol/L; 1 h, ≥ 10.0 mmol/L; and 2 h, ≥ 8.5 mmol/L^30^. The following criteria were used to identify women with preeclampsia: systolic blood pressure ≥ 140 mmHg and/or diastolic blood pressure ≥ 90 mmHg after 20 weeks of gestation in previously normotensive women, along with the presence of proteinuria (two or more dipstick readings of 2+ or greater, one catheter sample reading of 1+ or greater, or a 24-h urine collection containing at least 300 mg of protein)^31^. Blood pressure was measured on the right upper arm after a 5–10 min rest in a quiet environment by the mercurial blood pressure device (HEM-7117, OMRON, Japan). Cesarean deliveries performed under any condition were included.

### Measurement of neonatal outcomes

Information of body weight and length of children were drawn from the medical birth registries. Anthropometric measurements of neonates were conducted by trained medical workers in each study center according to standard procedures. Recumbent length of children were measured to the nearest 0.1 cm with a stadiometer, and weight was assessed to the nearest 100 g. Preterm birth was defined as < 37 weeks of gestation^32^. Stillbirth was defined as the death of the fetus after 24 weeks of gestation ^33^. A body weight of over 4000 g regardless of gestational week was considered as fetal macrosomia^34^. Small and large for gestational age at birth were defined as sex- and gestational age-adjusted birth weight < 10th percentile and > 90th percentile, respectively^35^.

### Statistical analysis

The influence of pre-pregnancy BMI (WHO Asian BMI categorization) on related diseases [gestational diabetes mellitus, preeclampsia, cesarean delivery, preterm delivery, stillbirth, macrosomia, small for gestational age (SGA), and large for gestational age (LGA)] were examined by odds ratios and 95% confidence intervals estimated using logistic regression analysis (P values correspond to Wald test).

For calculation of adjusted odds ratio of BMI with stratification according to maternal age (< 25 years, 25–29 years, 30–34 years, > 35 years), multiple logistic regression models were adjusted for maternal age, gestational weight gain, parity, gestational age, infant’s sex, and maternal marital status. Models for cesarean delivery, preterm delivery and stillbirth were additionally adjusted for GDM and preeclampsia. The optimal model selection was performed by using the backward stepwise procedure with Akaike's Information Criteria (AIC) as evaluation criteria.

In addition, variable importance based on the gradient boosting machine^36^ was used to assess the influence of pre-pregnancy BMI (continuous variable) and material age (continuous variable) for adverse pregnancy outcomes. GDM and preeclampsia were additionally used in the gradient boosting machine for cesarean delivery, preterm delivery, stillbirth, macrosomia, SGA, and LGA. The maximum number of base learners for the gradient boosting machine was set to 2,000, and the optimal number of base learners was selected using the 10-hold cross-validation.

All data were analyzed using R version 3.6.0 (The R Foundation for Statistical Computing) and JMP Pro version 13.0.0 (SAS Institute Inc.), at a two-tailed alpha level of 0.05.

## Data Availability

To learn more about this cohort and explore potential collaborations with data, please contact the principle investigator of this study: Prof. Guo Cheng: (gcheng@scu.edu.cn).
